# Understanding why racial/ethnic inequities along the HIV care continuum persist in the United States: a qualitative exploration of systemic barriers from the perspectives of African American/Black and Latino persons living with HIV

**DOI:** 10.1186/s12939-023-01992-6

**Published:** 2023-08-30

**Authors:** Prema Filippone, Samantha Serrano, Stephanie Campos, Robin Freeman, Sabrina R. Cluesman, Khadija Israel, Brianna Amos, Charles M. Cleland, Marya Gwadz

**Affiliations:** 1 New York University Silver School of Social Work, 1 Washington Place North, New York, NY 10003 USA; 2 Division of Biostatistics, Department of Population Health, New York University School of Medicine, New York, NY 10016 USA

**Keywords:** Qualitative, HIV care continuum, racial/ethnic inequities, Systemic barriers, Structural barriers, Systemic racism, Structural racism

## Abstract

**Background:**

Racial/ethnic inequities along the HIV care continuum persist in the United States despite substantial federal investment. Numerous studies highlight individual and social-level impediments in HIV, but fewer foreground systemic barriers. The present qualitative study sought to uncover and describe systemic barriers to the HIV care continuum from the perspectives of African American/Black and Latino persons living with HIV (PLWH) with unsuppressed HIV viral load, including how barriers operated and their effects.

**Methods:**

Participants were African American/Black and Latino PLWH with unsuppressed HIV viral load (N = 41). They were purposively sampled for maximum variability on key indices from a larger study. They engaged in semi-structured in-depth interviews that were audio-recorded and professionally transcribed. Data were analyzed using directed content analysis.

**Results:**

Participants were 49 years old, on average (SD = 9), 76% were assigned male sex at birth, 83% were African American/Black and 17% Latino, 34% were sexual minorities (i.e., non-heterosexual), and 22% were transgender/gender-nonbinary. All had indications of chronic poverty. Participants had been diagnosed with HIV 19 years prior to the study, on average (SD = 9). The majority (76%) had taken HIV medication in the six weeks before enrollment, but at levels insufficient to reach HIV viral suppression. Findings underscored a primary theme describing chronic poverty as a fundamental cause of poor engagement. Related subthemes were: negative aspects of congregate versus private housing settings (e.g., triggering substance use and social isolation); generally positive experiences with health care providers, although structural and cultural competency appeared insufficient and managing health care systems was difficult; pharmacies illegally purchased HIV medication from PLWH; and COVID-19 exacerbated barriers. Participants described mitigation strategies and evidenced resilience.

**Conclusions:**

To reduce racial/ethnic inequities and end the HIV epidemic, it is necessary to understand African American/Black and Latino PLWH’s perspectives on the systemic impediments they experience throughout the HIV care continuum. This study uncovers and describes a number of salient barriers and how they operate, including unexpected findings regarding drug diversion and negative aspects of congregate housing. There is growing awareness that systemic racism is a core determinant of systemic barriers to HIV care continuum engagement. Findings are interpreted in this context.

## Introduction

The present study takes a qualitative approach to explore the perspectives of African American/Black and Latino persons living with HIV (PLWH) on systemic barriers to engagement along the HIV care continuum. Despite substantial progress in reducing HIV incidence and improving HIV treatment outcomes in the past decade in the United States, racial/ethnic inequities in engagement along the HIV care continuum – that is, in rates of HIV diagnosis, linkage to and retention in HIV care, HIV medication uptake, HIV medication adherence, and HIV viral suppression – are serious and persistent [1]. The long-standing patterns of insufficient engagement among African American/Black and Latino PLWH, including unacceptably low rates of sustained HIV viral suppression, have a number of serious public health repercussions, such as an elevated risk of HIV transmission to others, a greater prevalence of chronic comorbid diseases and related complications, and higher mortality rates [1, 2]. Thus, similar to other health conditions, African American/Black and Latino PLWH bear a disproportionate burden of morbidity and mortality compared to White PLWH [3].

There is a substantial literature on the factors that drive these inequities among African American/Black and Latino PLWH. The majority of studies on barriers to the HIV care continuum have focused on obstacles at an individual level of influence, and include domains such as insufficient behavioral skill, self-efficacy, and/or motivation to adhere to medications; medical distrust; depression; substance use; and experiences of stigma [4, 5]. There is a smaller literature on the role of systemic and structural factors that impede engagement along the HIV care continuum for this population of PLWH. We define systemic barriers as those that involve entire complex systems, such as political, legal, education, economic, health care, and criminal justice systems, and that create and maintain inequities [6–9]. Structural barriers are defined as the policies, institutional practices, and social norms that serve as the framework of the system [6–9]. Since structural barriers are aspects of systemic barriers, we use the term systemic barriers to refer to both concepts in this paper [6]. Indeed, there is growing recognition that the health of social groups is most strongly affected by systemic, rather than individual, phenomena [3]. Systemic barriers to HIV care continuum engagement have been documented in past research and include factors such as living in a geographical area with socioeconomic disadvantage; income maintenance support levels that keep people in poverty; lack of easily available high-quality services and housing; lack of integrated health systems and poor access to comprehensive, coordinated HIV care; and poor transportation infrastructures that impede access to care [7–9]. However, American/Black and Latino PLWH’s perspectives on systemic barriers have rarely been documented, a gap the present study addresses.

The public health system in the United States has mobilized to support the engagement of PLWH along the HIV care continuum. Federal government investment in the domestic response to HIV in service, treatment, and research domains has exceeded $28 billion per year [10]. With these funds, federal and local governments plan and manage safety net services for PLWH. This is vital because people at the lowest socio-economic strata are greatly over-represented among the population of PLWH [11, 12]. Low socioeconomic status places people at risk for contracting HIV, and living with HIV commonly pushes PLWH to a lower or low socioeconomic status position for a variety of reasons including staying unemployed because of concerns about loss of disability income benefits or of publicly-funded health insurance, and fears of discrimination in the workplace [13]. These studies suggest that for many if not most PLWH, poverty pre-dates the HIV diagnosis. Recent policy changes (namely, the Affordable Care Act) have improved shortfalls in medical insurance coverage and health care access for PLWH, including those assisted by the AIDS Drug Assistance Program (ADAP), which provides medications for low-income PLWH [14]. Further, stable housing is a well-known determinant of favorable HIV treatment outcomes [15] and federal programs fund housing options for low-income PLWH. Housing options for PLWH include permanent supportive housing, both congregate facilities that provide on-site supportive services and individual scattered-site apartments. While these housing programs improve HIV outcomes and wellbeing [16, 17], little is known about American/Black and Latino PLWH’s *experiences* in various housing arrangements.

Thus, even with essentially universal access among PLWH to HIV primary care and HIV medication along with social safety net programs, serious racial/ethnic inequities in HIV treatment engagement and outcomes persist [18–20], in large part, the literature suggests, due to systemic factors. The present study seeks to extend the existing literature on systemic barriers by uncovering and describing the perspectives of those who have the greatest challenges to consistent engagement along the care continuum, American/Black and Latino PLWH from the lower socioeconomic strata.

## Methods

### Overview

The present qualitative study uses data from a larger project, a pilot intervention optimization trial that took place between September, 2020 and January, 2022 in New York City. That trial was carried out virtually, because in-person activities with human subjects were prohibited at our institution due to COVID-19 restrictions. The trial enrolled 80 African American/Black and Latino PLWH who did not evidence HIV viral suppression based on a laboratory report. It used a factorial design to explore the effects of three behavioral intervention components and the primary outcome was HIV viral suppression. The pilot optimization trial is described in detail elsewhere [21]. A subset of participants in the optimization trial engaged in in-depth, qualitative semi-structured interviews (N = 41). The goals of the present study are to use these qualitative data to uncover and describe participants’ perspectives on systemic barriers to the HIV care continuum. The study was approved by the Institutional Review Board at New York University. Participants gave verbal informed consent for study activities.

### Recruitment for the optimization trial

Participants were recruited using a hybrid method that included peer-to-peer referral, flyers posted in local community-based organizations, and advertisements placed in the medical research section of a local free newspaper.

### Eligibility criteria for the optimization trial

Inclusion criteria were: aged 18–65 years, diagnosed with HIV, lives in the New York City area, able to engage in research in English, can receive text messages on a phone, had not participated in an economic incentive program for HIV medication adherence in the past month, was not a participant in either of two recent research studies carried out by this team, can provide a recent laboratory report showing levels of HIV viral load within the past two months, and the laboratory report indicates unsuppressed HIV viral load (≥ 200 pp/mL). Race/ethnicity were not eligibility criteria, but it was anticipated that > 90% of those screened would be African American/Black or Latino given demographic characteristics of PLWH in the local area (> 75% African American/Black or Latino; [22, 23]). All participants enrolled were either African American/Black or Latino.

### Procedures for the present study

From those enrolled in the optimization trial, we recruited participants for qualitative in-depth interviews at two time points. Participants were purposively sampled for maximum variability on key indices including HIV viral suppression (yes or no), sex assigned at birth, and years since HIV diagnosis. The interviews lasted between 60 and 90 min. Due to COVID-19 restrictions, they were conducted over the phone. A semi-structured template was used by trained interviewers to guide the interviews. Interviews were audio-recorded and then professionally transcribed. The first set of interviews was conducted four months after enrollment and the second was conducted eight months after enrollment. Those who engaged in the first interview were not recruited for the second. We interviewed 21 participants for the first assessment and 20 for the second (total N = 41). Interviews were carried out by two experienced PhD-level qualitative researchers who were trained as anthropologists (a medical anthropologist and cultural anthropologist). Participants were compensated $30 for the interview. Compensation was provided using the Greenphire ClinCard system, a refillable debit card system for research studies. The present study also draws on quantitative data collected at the baseline assessment to describe the sociodemographic and background characteristics of the sample. We describe those measures elsewhere [21].

### Theories guiding the present study

The present study was grounded in an integrated conceptual model developed by our team that combines critical race theory, harm reduction, and self-determination theory, as described in more detail elsewhere [24, 25]. This integrated conceptual model aligns with the theory of triadic influence, a social-cognitive theory that highlights the relevance of simultaneous systemic/structural-, social-, and individual/attitudinal-level streams of influence on behavior [26], as well as highlighting the importance of structural and cultural competency in health care settings. Structural competency is the ability to determine how issues defined as symptoms, attitudes, or diseases, such as poor engagement along the HIV care continuum, actually represent the downstream repercussions of powerful upstream influences including health care delivery systems, federal benefits policy, and transportation infrastructure [27]. Cultural competency describes the ability of systems to provide care to clients with diverse values, beliefs, and behaviors, including the tailoring of health care delivery to meet patients’ social, cultural, and linguistic needs [28]. The integrated conceptual model further emphasizes the importance of approaches that support individual autonomy and harm reduction as aspects of structural and cultural competency in health care settings.

### Qualitative interview template

The semi-structured interview template was developed by the research team, which included experts on poverty, sexual/gender minority status, African American/Black and Latino PLWH, and the HIV care continuum. The template and the analyses reflected the integrated conceptual model described above. The template was flexible and structured as a sequence of questions and related prompts. It moved from general to more specific questions and was divided into two parts. The first part of the template focused primarily on views on the behavioral intervention components participants received as part of the optimization trial. The second part of the template focused primarily on the context of HIV management and experiences in the optimization trial more generally. The present study does not focus on the optimization trial specifically, but the questions in the interview template yielded detailed information on HIV management and participants’ larger life contexts. We analyzed these data for the present study. Questions in the interview template included: a general overview of the participant’s experience in the project, called S-CAP2 (e.g., What stands out to you most about the S-CAP2 project?); views on sustaining HIV viral load, where relevant (e.g., Do you plan to continue taking HIV medication and/or sustain an undetectable viral load after the [study ends]? Why or why not?); acceptability, feasibility, and effects of the larger study (e.g., Has there been anything about S-CAP2 that you think has been particularly unhelpful? Helpful? What do you think should be included that was not included?); and 4) aspects of COVID-19 (e.g., Looking back, in what ways did the COVID pandemic influence your HIV management?). Prompts in some cases focused on participants’ views on race and racism, and also allowed for participants to introduce these and related constructs in their interviews, consistent with the qualitative and exploratory approach.

### Data analyses

Analyses were led by the two qualitative researchers who carried out the in-depth interviews. They were assisted by an interpretive community of research team members, which included cisgender men and women, transgender, gender non-binary, or gender-fluid individuals, heterosexual and gay/lesbian/bisexual/queer individuals, and people from White, African American/Black, mixed race, Asian, and Latino backgrounds [29, 30]. Data were managed and analyses were carried out in the Dedoose platform. We took a directed content analysis approach that was both inductive and theory-driven [31]. We began with an initial list of “start codes” along with their operational definitions that was generated by the primary qualitative analysts. This initial start code list was informed by the theories and perspectives framing the study; namely, the integrated conceptual model [24, 25] and theory of triadic influence [26]. Codes were generated that reflected potential systemic barriers (e.g., housing circumstances, poverty), culture and race/ethnicity (e.g., experiences of discrimination and racism, medical distrust), substance use management, autonomy, competence, relatedness, and other factors known to promote or impede engagement along the HIV care continuum (e.g., mental health distress). Using this scheme, the primary analysts coded interview transcripts. During the coding process, codes were refined, clarified, and/or broadened; for example, when new codes were identified (e.g., quality of housing, diverting HIV medications). Discrepancies in codes and coding between the data analysts were resolved through in-depth discussion and negotiated consensus. This approach to resolving discrepancies is commonly used in the published literature [32]. Then, the interview transcripts were recoded using the final coding frame. Codes were then combined into larger themes and sub-themes in an iterative process led by the two main data analysts and in collaboration with the interpretive community. We wish to note that although participants were certainly influenced by multiple, intersectional systems of oppression related to race/ethnicity, socioeconomic status, sex, sexual orientation, gender identity, and HIV status, we did not attempt to analyze data from an intersectional perspective due to the modest sample size. Instead, we organized codes into themes that applied to the sample as a whole, focused on participants as African American/Black or Latino PLWH from low-socioeconomic status backgrounds who did not evidence HIV viral suppression at the time they entered the larger study.

Methodological rigor of the analysis was monitored continually in several ways. An audit trail of process and analytic memos was maintained [33]. Analysts engaged in debriefing sessions approximately monthly with the interpretive community. The primary analysts and the interpretive community attended to the potential effects of the team’s positionality related to power and privilege, sex, gender, sexual orientation, race/ethnicity, health, and socioeconomic status throughout the data collection process through reflection and training that focused on how these factors might affect interviewing and data analytic processes [34, 35].

## Results

Participants were 49 years old, on average (SD = 9 years), 76% were assigned male sex at birth, 34% were sexual minorities (lesbian, gay, bisexual, queer, non-heterosexual), and 22% had a gender identity that was transgender, gender-nonbinary, gender non-conforming, or gender fluid (Table 1). Most were African-American/Black (83%) and the remainder were Latino. A total of 66% had completed high school or equivalent secondary education. Almost all (93%) had been homeless in their lifetimes and close to half (46%) had been homeless in the past year. Nearly half (49%) resided in their own home or an apartment that they rented or owned, including scattered-site permanent supportive housing, 29% lived in a single room occupancy residence (congregate facility housing), 7% in a shelter, and the remainder in some other type of setting. Regarding indications of poverty or extreme poverty, all received public assistance, most had indications of food insecurity (86%), only 2% were employed, and half (51%) ran out of funds for basic necessities at least monthly in the past year. Other sociodemographic and background characteristics are presented in Table 1.

**Table 1:** Sociodemographic and background characteristics (N = 41)

	Mean (SD) or %	N
Age in years (M, SD)	48.5 (9.40)	
Median, [minimum, maximum], in years	52.0 [31.0, 62.0]	
*Sex assigned at birth*		
Male sex assigned at birth	75.6	31/41
Female sex assigned at birth	19.5	8/41
Other sex assigned at birth	4.9	2/41
*Sexual orientation*		
Sexual minority (lesbian, gay, bisexual, queer, non-heterosexual)	34.1	14/41
*Gender identity*		
Transgender, gender-nonbinary, or gender fluid gender identity	22.0	9/41
*Race/ethnicity*		
African American/Black (non-Latino/Hispanic)	82.9	34/41
Latino	17.1	7/41
*Education*		
High school graduate/equivalent or higher	65.9	27/41
*Past and present homelessness*		
Homeless over the lifetime	92.7	38/41
Homeless in the past year	46.3	19/41
*Current housing circumstances*		
In own home or apartment (rented or owned, including scattered-site permanent supportive housing, but not congregate facility housing)	48.8	20/41
Single Room Occupancy residence (SRO; congregate facility housing)	29.3	12/41
Shelter (temporary residence for homeless individuals and families)	7.3	3/41
Other type of residence	14.6	6/41
*Indications of poverty*		
Receives public assistance	100	41/41
At least one indication of food insecurity	86.3	35/41
Currently employed full- or part-time	2.4	1/41
Ran out of funds for basic necessities monthly or more in past year	51.2	21/41

As shown in Table 2, participants had been diagnosed with HIV for an average of 19 years (SD = 9 years). Almost all (95%) had taken HIV medication in the past. The majority (76%) had taken HIV medication in the four weeks prior to enrollment, although not at levels sufficient to reach HIV viral suppression. Self-reported adherence on a visual analog scale ranging from 0 to 100 was sub-optimal (mean = 60 points, SD = 37 points). Almost all (98%) were covered by public health insurance (e.g., Medicaid) and the remainder were uninsured. 42% of participants evidenced HIV viral suppression during the optimization trial. Alcohol use at a moderate-to-high risk level was common (61%), and less than half used cannabis (44%) or cocaine (32%) at a moderate-to-high risk level. Less than half (42%) evidenced polysubstance use at moderate-to-high risk levels, that is, two or more substances other than tobacco or alcohol. Most (73%) had been in substance use treatment in the past.

**Table 2:** Health factors (N = 41)

	Mean (SD) or %	N
Years living with HIV/years since HIV Diagnosis (M, SD)	18.8 (8.93)	
Median, [minimum, maximum], in years	19.0 [1.00, 37.0]	
Perinatally infected with HIV	0.0	0/41
Has taken HIV medication in the past	95.1	39/41
Years since first initiated HIV medication	16.7 (8.74)	
Median, [minimum, maximum], in years	15.5 [1.00, 38.0]	
Number of HIV medication starts (range 0–288 times)	6.08 (7.93)	
Longest duration of sustained HIV medication over the lifetime, in months	34.3 (41.6)	
Median, [minimum, maximum], in months	24.0 [1.00, 240]	
Took at least one dose of any HIV medication in the past 4 weeks	75.6	31/41
Adherence to HIV medication - past month (range 0–100)	59.9 (37.0)	
If not on any HIV medication at enrollment, number of months since last dose	6.40 (5.64)	
Median, [minimum, maximum], in months	4.10 [1.50, 18.4]	
Is covered by public health insurance (e.g., Medicare, Medicaid)	97.5	40/41
Satisfaction with HIV care (range 0–100)	76.8 (23.6)	
Evidenced HIV viral suppression at follow-up	41.5	17/41
*Substance use*		
Alcohol Use at a Moderate-to-High Risk Level	60.9	25/41
Cannabis Use at a Moderate-to-High Risk Level	43.9	18/41
Cocaine Use at a Moderate-to-High Risk Level	31.7	13/41
Polysubstance Use (2 + substances excluding tobacco and alcohol) at a Moderate-to-High Risk Level	41.5	17/41
Any Substance Use Treatment Lifetime	73.2	30/41

**Overview**. Participants were highly motivated to maintain optimal health, although this did not always involve, in their views, taking HIV medication consistently or sustaining HIV viral suppression. Overall, they understood, accepted, and acknowledged the importance of adhering to HIV medication and reaching HIV viral suppression (also called undetectable viral load) as critical aspects of HIV management. Thus, they did not on the whole evidence major gaps in the extent of their knowledge regarding HIV treatment. Notably, this stance on the importance of HIV medication commonly co-occurred with fears and concerns about the medications’ side effects and with distrust of HIV medications. Thus, findings indicated that HIV knowledge, attitudes, beliefs, and emotions were tightly intertwined. Further, in addition to HIV knowledge, participants generally expressed the desire or intention to take HIV medication with high levels of adherence and to reach HIV viral suppression, at least in theory, either at the present time or in the future as conditions allowed. Some participants had past periods of HIV viral suppression, indicating they had gained the behavioral skills to adhere to medication. In this context of what can be described as generally high motivation to take HIV medication with high levels of adherence, with some ambivalence, and the skills to do so, they enumerated a number of serious systemic challenges, both from their current circumstances and the past. We organized codes into one primary theme and four related subthemes. The primary theme described serious, chronic poverty as a fundamental cause of insufficient HIV management, suboptimal physical and mental health generally (including depression), and poor quality of life. Indeed, the centrality of chronic poverty in the lives of participants cannot be over-stated. Specifically, financial insecurity and insufficiency resulted in participants’ inability to meet basic needs, and these basic needs were necessarily prioritized over HIV management. Related to this primary theme, we identified the following inter-related subthemes describing specific systemic factors impeding HIV management, all of which were rooted in or exacerbated by chronic poverty; namely, the effects of housing stability or instability, type, and quality, including the adverse effects of congregate care on health and wellbeing (an unexpected finding given the literature on the benefits of housing placements for PLWH); generally positive experiences with HIV care providers, with some sources of tension (e.g., related to substance use), which suggested that insufficient structural and cultural competency among providers was fairly common, and further, health care settings were generally challenging to navigate; and pharmacies commonly offered to provide participants with cash if they did not fill their prescriptions, called “drug diversion”. Soliciting drug diversion is a type of insurance fraud on the part of the pharmacies and is illegal [36]. The pervasiveness of drug diversion in this sample, both presently and in the past, and in participants’ social networks, was another unexpected finding. Last, COVID-19 exacerbated structural factors and mental health and substance use concerns. The constructs of race, racism, and discrimination were not generally mentioned in the interviews, even when participants were directly queried.

Participants reported being faced with these systemic impediments on a daily or almost daily basis, and described these factors as overwhelming and insurmountable. Yet, at the same time they grappled with these systemic factors, and had periods of successfully mitigating the adverse effects of chronic poverty and its related challenges. That is, they evidenced strengths and resilience. But the context in which participants were expected to manage HIV was clearly difficult and not sufficiently conducive to health and wellbeing, as participants described below. These challenges were present before the COVID-19 pandemic, but the pandemic generally made systemic barriers substantially more difficult to circumvent, and also introduced new obstacles. Further, systemic barriers were inter-related. Names presented below are pseudonyms and some identifying details have been changed or obscured to protect confidentiality. Participants’ preferred pronouns were used in their descriptions.

### Chronic poverty, financial insecurity, and financial insufficiency

Participants noted that the inability to meet basic needs for food, clothing, toiletries, and other necessities made it exceedingly difficult to focus on anything else, much less HIV medication adherence. Maya was a 60-year-old Black, heterosexual, cisgender woman who was diagnosed with HIV when she was in her mid-40s. She described being referred to a set of services and resources by a social service organization, but had trouble accessing services because of the complexities of her needs in the context of chronic poverty, and the inability to use Telehealth to access care:

I was low on food. I needed clothing like shoes. [...] I never got to the place [I was referred to] but I still have it on my phone if I need to go. They [the social service organization] gave me a lot of information. I was reading through it. But sometimes I have difficulty reading. Not reading but concentrating. […] It’s frustrating. I’m on psych meds, but I haven’t been taking them like I’m supposed to because of […] the corona virus. But anyway, I’m trying to get back on track. […] But I couldn’t get to my doctor, I had to do video teleconference and my phone was all messed up. […] I’ve been losing stuff lately because I forget, and then my hands aren’t working properly. I have difficulty with my fingers. […] It’s a lot. It’s a whole lot that I’m trying to cover at one time. But I’m doing OK. I’m getting better. I’m getting a lot better because I was worse than this.

Moreover, Maya had relied heavily on a home health aide to support HIV and psychotropic medication adherence, but these services were disrupted during the COVID-19 pandemic. Yet, Maya was certain the services would re-start soon, and said, “After that hopefully we’ll get back in our regimen.” Maya’s experiences highlighted the numerous frustrations and cascading challenges she faced navigating the systems she relied on in the context of poverty (phone problems impeded access to psychotic medication which contributed to confusion which fostered food insecurity), similar to most other participants, along with a sense of her coping abilities, optimism, and resilience.

For many participants, the inability to afford basic necessities forced them to make difficult decisions about their health, including whether or not to sell their HIV medications to pharmacies that solicited the prescription (as described in more detail below) or take the HIV medication. Byron, a 35-year-old Black, sexual minority, cisgender man who was diagnosed with HIV when he was under the age of 18 years, had the following to say:

But I really, I need the money [from the pharmacy]. I don’t get SSI [Supplemental Security Income] and this little money they give me for public assistance is not enough to take care of me for a month. Negative. It’s not enough. [...] The fact of I need to take my meds or I’m going to die. Or it’s either choosing to take the meds and stay financially twisted, because that $350 [from the pharmacy] is my biggest check in the month – that’s my biggest check, is the medicine I sell. [...] I need my money so I can just eat and do my regular household stuff and my laundry and my restaurants that I eat at. […] The hard part is to stop [selling]. […] It’s so hard.

Thus, we found the effort, creativity, and resourcefulness needed to meet basic physical and emotional needs necessarily took precedence over HIV care and HIV medication adherence. Nonetheless, participants frequently described adaptive strategies to cope with chronic poverty. These adaptive strategies included taking advantage of food pantries and other resources and services, cutting back on basic necessities, and participating in various health-related research studies that provided financial incentives, along with selling HIV medication when necessary and when pharmacies initiated these transactions. Yet, meeting basic needs was time-consuming and experienced as dignity-denying, exhausting, and stressful. Further, chronic poverty also fostered poor-quality or unstable housing circumstances, as described in the next section.

### The effects of housing stability, type, and quality

Participants generally received housing assistance through programs for PLWH administrated by the local department of health (e.g., permanent supportive housing or long-term rental assistance) or for those unhoused (e.g., shelters for homeless individuals or families), although some obtained housing independently. Some participants lived in congregate facility housing such as single-room occupancy residences (SROs), a form of housing comprised of small, furnished single rooms generally without private kitchens or bathrooms. Others resided in temporary locations such as emergency shelters, where securing a bed each night proved remarkably difficult, or stayed temporarily with friends and family. Even when stable, private housing was obtained, financial challenges often precluded participants from staying there long. Thus, despite the array of housing services available to PLWH, housing circumstances were constantly changing, under threat, or inadequate. These congregate living conditions had a number of characteristics that created serious obstacles to health and wellbeing; namely, a lack of personal safety, privacy, and autonomy; limited or no access to essential resources such a private kitchen; continuous emotional stress; social isolation; and circumstances that created inconsistent access to medications. The impediments to HIV management that arose from these adverse housing conditions were generally experienced by participants as insurmountable, even in conjunction with strong motivation to take HIV medication consistently. Rhonda, a 55-year-old Black, heterosexual, cisgender woman who was diagnosed with HIV when she was in her mid-20s, for instance, directly attributed her inability to consistently take HIV medication to losing her apartment and being forced to move constantly back and forth between various emergency shelters. Rhonda recalled the following:

I was taking [HIV] meds and I wasn’t really, because like I said I was in the psych ward, then […] into shelters after shelters after I lost my apartment so I wasn’t really keeping up [with HIV medication]. […] This is supposed to be my apartment now because I’m off the shelters, so I’m in my apartment and I’ve started back like daily taking my medication because I was undetectable for like five years straight, four years straight when I was in my apartment. Then when I lost my apartment, going from shelter to shelter, I slacked up in taking my medication, you know? Then I was back in my own and I could start back taking my medication again.

Notably, taking HIV medication and reaching HIV viral suppression was possible only after Rhonda was housed in a stable, private setting.

Participants commonly described their congregate housing circumstances as unsanitary, chaotic, and physically dangerous, and noted that these conditions exacerbated mental health issues, which in turn negatively affected their ability to focus on health, including taking HIV medication. Participants described illicit substance use as ubiquitous among fellow residents in congregate settings. This contributed to participants’ frustration with their housing conditions, especially for those seeking to avoid using substances at all or intending to use substances socially or at non-hazardous levels. Relationships in these congregate settings were described as largely transactional in that social interactions most commonly took place when a fellow resident wanted or needed something, including to sell or purchase drugs. This congregate environment, paradoxically, contributed to social isolation. As Maya, introduced above, noted:

Because I live in a place [supervised living] where there’s a lot of people that just all over the place. There’s people running around all day and all night. And they’re annoying. […] So, they’ll sleep all day and then nighttime they’re up. […] Somebody will start banging on the wall. People start yelling in the hallway. People will knock on my door. So sometimes it becomes very, very tough, annoying. […] I’ve never lived in a place like this my entire life. […] It affects me because it stops me from taking my meds. […] And then when you open up the door, then it’s a whole bunch of nonsense I have to deal with. Then if I don’t open the door, they stand right in front of my door, and I have to start hollering at them and yelling at them to get away from my door. And I don’t like that because it takes up time, it stressed me, it takes up time for me doing the things I need to do. Then I have to calm down. It’s crazy.

Mannie was 60 years old and identified as heterosexual, transgender, and Latino, diagnosed with HIV over 30 years before. (Mannie used they/them pronouns.) They described their struggles with substance use in the context of a partner who used drugs and being located in a harm reduction housing program. They noted:

I went in there five and half years clean, and I was there not even six months and I relapsed. Which sucks. […] It’s not a good lifestyle. […] I go through a lot with my partner. We’re together nine years and it’s hard to stay clean when all he wants to do is smoke crack. […] And that’s [drug use] all around in my building, the neighbor next door, the neighbor across the hall. And I’ve been telling these people [caseworkers] to help to get me out of there, like give me some help, some – a push in the right direction. And even my [caseworker], my methadone counsellor, supposedly they’re both working on it together to try to get me out of that building. Because if I go to rehab – I’m not going to go to rehab and come back to that building, because they know how I am. And I’ve seen it happen to too many other people where they go to [rehab], get clean, and then they come back [to the housing placement], and then they use again, and they’re back where they started.

While harm reduction is a critical aspect of substance use treatment for PLWH, it was not necessarily optimal for Mannie at this time. Other participants drew a direct connection between the lack of privacy and autonomy in their current congregate living situations and their abilities to prioritize HIV medication adherence. Asked specifically how improvements in his housing circumstances affected his ability to consistently take his HIV medication, Cameron, a 55-year-old Black, heterosexual, cisgender man who was diagnosed with HIV in his mid-30s, responded as follows:

When you ain’t got to share kitchen and bathroom, like our microwave was in our room. So now being that you’ve got everything in your room, it’s more better for you, more healthier. You know? It’s more better, more healthy living. You can think better.

However, it took Cameron sixteen months to move from a shelter to this private setting, and he was not taking HIV medication consistently during that waiting period. Taken together, these findings described how improved and high-quality living conditions facilitated HIV medication adherence, and physical and mental health more generally. As Jamie, a 30-year-old Black, sexual minority, cisgender man who had been living with HIV for over 15 years, said, “I know for one thing being because [since] I’ve been in my apartment, my outlook on things have changed.”

### Mixed experiences with providers and strained experiences with settings

We found participants’ relationships to health care providers were mostly positive, albeit with sources of tension, and also that relationships to health care settings tended to be strained and difficult. Those mostly satisfied with their health care providers highlighted the importance of the providers’ supportive and non-judgmental stance. However, one source of frustration for participants regarding providers had to do with a mis-match between how they managed their HIV medication (i.e., taking medication every other day instead of every day) with providers’ expectations and recommendations (i.e., take every dose, every day). Providers were described as being concerned about participants’ health and about their developing resistance to medications, which participants acknowledged were legitimate concerns. But participants also had apprehensions about the toxicity and other adverse effects of HIV medications. It was common for participants to describe how providers did not acknowledge participants’ own expertise about their health, lived experience, personal decisions, and distrust of and doubts about HIV medications, and instead maintained their stance that medications should be taken every day. In some cases, participants reached undetectable HIV viral load levels on their idiosyncratic dosing schedules. In fact, many HIV medication regimens are highly effective at less than 100% adherence [37]. In other cases, participants did not reach unsuppressed HIV viral load levels on their idiosyncratic dosing schedule but were able to reduce viral load levels. However, participants reported providers did not engage participants in discussions of their fears and dosing schedules. Some participants stopped and started HIV medications as a means of managing perceived medication toxicity, a pattern that is not recommended for immune health [38]. Yet, fear and distrust of HIV medications have systemic and cultural roots [39], and explicit acknowledgement of these roots would be an indication of structural and cultural competency on the part of providers. Warren was 35 years old and identified as Black, gender non-conforming, and sexual minority, and was diagnosed with HIV in their mid-teens. (Warren uses they/them pronouns.) They noted the following:

Well, I was [taking breaks from my HIV medication] before I told my doctor. So, I was already in the process [of taking breaks] for a good three months. And I told her. She was like, “Well sorry, that’s how you can become resistant and all this and all that.“ But I had it [HIV] since [I was] 15 […] and I’ve been on […] like three different ones. And when she did my resistance thing, I wasn’t resistant to anything. Do I trust that I should take it every day? I do not because at the end of the day anything we take that’s not growing out of the ground or got its head chopped off is a poison. I just feel like once I am undetectable, I fall back for a little bit. Because my body is filtering out that poison every day, every day, it’s going to catch up sooner or later. Don’t they say nowadays people don’t die from AIDS; they die from complications from taking the medication?

Warren highlighted the complexity of HIV management in the context of fear of HIV medications and medical distrust, and also that distrust and fear can co-occur with taking medication. However, Warren noted their provider did not engage in a discussion with them about their fears, distrust of medication, and reasons for stopping and starting HIV medication, but instead provided what Warren considered standard medical advice. Further, Warren pointed out a pattern found among this population of PLWH stopping HIV medications after receiving test results indicating suppressed HIV viral load levels, also not recommended, highlighting the challenge of sustaining HIV viral suppression.

Substance use was another complex issue that participants and health care providers had to navigate. Overall, participants did not experience their health care providers as punitive with respect to their substance use. Providers commonly advised, recommended, and encouraged participants about non-hazardous substance use management and avoided stigmatizing language and behaviors, and participants in turn appreciated this mostly non-judgmental and non-stigmatizing stance. Still, despite the prevalence of substance use in participants’ lives, providers generally took an abstinence-only approach and thus harm reduction approaches were lacking. Andrew, a 50-year-old Latino, heterosexual, cisgender man diagnosed with HIV 20 years ago, noted:

I go pretty much to the same doctors and – I switched to [health care setting] a while back and it’s pretty good. They really seem to really care what you’re doing because they’re like HIV specialists. […] I’ve given urine lots of times and we talk about it, and he says, “[Andrew], you’ve got to stop with this cocaine. It’s not good for you blah blah blah.” They just seem to care about me. They never threatened to cut off the [HIV] medicine.

While Andrew experienced his provider in a positive light, his quote highlighted the provider seeming to take an abstinence-based approach to substance use, rather than including options for harm reduction, which may have given Andrew a broader range of strategies to manage use and reduce harm from use. Further, Andrew’s quote implied a concern that he could in fact be cut off from HIV care due to his cocaine use and/or that other settings had done so or could. Yet, as noted above by Mannie (who resided in a harm reduction housing setting), harm reduction was not useful for all participants, highlighting the need for open communication with social service and medical providers about substance use and an individualized approach in HIV care settings.

While Andrew did not experience substance use as an impediment to engaging in HIV care, it did in fact commonly serve as a barrier for participants, in part related to how providers discussed drug use with their patients. Wynn, a 60-year-old Black, heterosexual, cisgender woman who had been living with HIV for half of her life, described the following:

I did feel was guilty when they took my blood work, and I had drugs in my urine. And a lot of times I didn’t want to go [to HIV care] because I knew I had used the day before. So, I would miss that doctor’s appointment, and wait for another [day]. […] It was a lot of headache trying to [schedule], because you never know when you’re going to use. No, now it don’t get in the way. I use. I’m still going to the doctor because I want to know what’s going on. He said, “It would be better not to use.“ He said, “Because it makes you a more positive person so that the medicine will work better.” Because by using drugs it interrupts stuff, and […] it [HIV] wasn’t clearing up because I continued to use.

Notably, while Wynn missed some appointments, she also attended others, but her quote indicates the challenges inherent in engaging in HIV care while using drugs between appointments, and the anxiety inherent in doing so, since drug use can interfere with health and the ability to receive HIV care. Similar to Andrew, however, Wynn did not receive detailed or nuanced information about substance use management, but instead it was recommended to abstain.

Warm and supportive relationships with HIV medical providers were fairly common and were vital in participants’ lives. In one instance, this encouraging relationship was motivation for regularly taking one’s HIV medication, as Marion, a 70-year-old Black, heterosexual, cisgender man who was diagnosed with HIV when he was in his late 30’s, noted:

When I went to go see my doctor, right, I hadn’t seen my doctor in like four months, and when I went to go see my doctor in that fourth month, all she could do was just like – she got emotional. […] My doctor was really loving and caring – you know, she retired now. And she was very emotional when I stopped taking them [HIV medication] and I [had been] doing so well. And she said to me, “Mr. [name redacted], let me explain something to you right now. Sometimes in life, situations happen. We have to learn how to battle with situations that come in our lives. And right now, you don’t need to be playing around with yours, because you just got off,” – I’ve just been sober, three years, off of drugs and then I stopped taking the [HIV] medication. She was like, “No, that’s not good.”

While relationships with health care providers tended to be generally positive, participants were generally frustrated with the healthcare system as a whole. For example, services were difficult to access and wait-times for appointments were long. Byron, introduced above, described his experiences with the health care system:

I’ve had major issues with the doctor in the past year. At my other hospital, she [health care provider] had got COVID back in the spring and went out on leave and never came back. So, I had to switch doctors. And as a result, during when the COVID first hit, most places wasn’t taking new patients yet, so I was receiving my care at [a storefront urgent care center]. And they don’t normally do HIV care. The reason why I left that [HIV] clinic is my husband. […] They were not giving him any services like they were giving to me. […] And so, I wind up flipping out and wind up getting discharged. I […] got knocked out of the [HIV care center]. […] And that was over a year ago.

### Pharmacies purchased HIV medications, a type of insurance fraud

Some participants described selling their HIV medications to pharmacies, either currently or in the past, along with the factors that motivated selling and how the process of selling operated. The typical scenario involved pharmacies offering to purchase participants’ prescriptions when they went to collect their medications, with amounts varying between $100 and $350 per bottle. The primary reason for participants selling medication was serious financial need. Overall, participants would have much preferred to use their HIV medication for themselves and not sell it. However, it was common for them to be unable to decline the offer to sell medications in times of financial hardship (e.g., after losing a job or when a partner lost his/her/their job) and/or in the context of mental health or substance use challenges. Selling medications appeared more likely among those believing they were in a good state of health and with recent optimal HIV management and favorable HIV indicators (i.e., low HIV viral load levels and high CD4/T-cell counts) compared to those with more concerning HIV indicators (i.e., high HIV viral load levels and low CD4/T-cell counts) and recent poor HIV management. In most cases, participants were not entirely comfortable with selling medication and stopping their regimens, but commonly determined that taking a break from medication was tolerable in light of past optimal HIV management and in the context of extreme financial need and the opportunity to sell medications. In other cases, participants were worried or fearful about selling medications and thereby stopping their regimens, describing anxieties about the long-term health effects of taking a break from HIV medication. Thus, selling medications was one reason people stopped and started HIV medications, which, as noted above, is not medically recommended. Social service and medical providers were aware that some pharmacies purchased HIV medications, and in some cases guided (or forced) participants to avoid these pharmacies.

Some participants sold HIV medication occasionally as needed (“If I need some money, I’m not going to lie, I’ll go sell a bottle here or there”). In other cases, participants sold HIV medications mainly in times of heavy substance use in order to provide funds to purchase illicit substances. Mental health challenges such as depression and heavy substance use reduced internal resources required to decline selling medications and at the same time find other ways to meet material needs. Other participants sold their HIV medication regularly to make ends meet, but not necessarily related to heavy substance use, and this money made up a large portion of their monthly income. Andrew, introduced above, highlighted how the need to address financial problems triggered by unemployment or to supplement low wages promoted drug diversion, in conjunction with positive immune functioning indicators which suggested that he could weather a period off of HIV medication. He described:

And I’d like to say no [to selling HIV medication], but it’s like, when T-cells are way up and I’m broke – see I was working at a [mail and shipping] store for six months – I was just fired. Now I’m getting this job – one of the guys at my church has an uncle who owns – who runs these carwashes on the streets, like a van with a big water tank and people stop and you wash their car. You still make between $150 and $180 a day. And I’m saying, “Well, that’s great. I was making $60 a day [before].” This is off the books – I’d rather do that, and I can’t imagine how washing cars is going to knock the crap out of me, too, but [laughs] I got to do something. This means a better way of making some money –better than that stupid crap like selling my medication.

Thus, Andrew was actively searching for ways to generate an income that would allow him to decline drug diversion, including taking on physically challenging work. But he highlighted how challenging it was to decline selling HIV medication in the larger context of financial instability and low wages. Selling medications often created stress and anxiety for participants who felt caught between financial need and the desire for maintaining high levels of adherence to HIV medications. Further, as noted above, some participants sold HIV medications to purchase illicit drugs. They described how their substance use patterns were a barrier to taking medication consistently, since HIV medication was sold with the goal of buying substances and selling, of course, prevented them from using the medication for themselves. Andrew, introduced above, shared the following:

There’s always the drug factor. I get high once in a while, you know – it doesn’t help because that’s a real attraction like, mentally, you know. I’m usually pretty good about taking it [my medication]. It [having the medication] just makes you more likely to sell it because, you know, that’s a few hundred bucks. But, you know, we get high a lot, so that’s a factor.

Participants also described why they stopped selling HIV medication. They noted their desire to maximize their health and the fear of punishment as reasons for no longer selling HIV medication. For example, they acknowledged that the risks to one’s health by staying off HIV medication for long periods outweighed the benefits of selling their medications, particularly as they aged. For example, Harold, a 60-year-old Black, heterosexual, cisgender man who was diagnosed with HIV when he was in his late teens, was mandated by his case worker to switch pharmacies as a means of preventing him from diverting HIV medication. On the other hand, if Harold did not switch pharmacies, he would lose access to his treatment program:

I had to change to a pharmacy that I knew didn’t pay [for HIV medications] and that would deliver [to the home]. My case worker here at the programming now, actually it was mandated that in order for me to come back I must switch pharmacies. […] They told me in order to come back you must switch. They actually did that for my health, they said, “Well you going to that pharmacy, using the pharmacy. Sometimes you sell your medications. So that pharmacy is really no good to you or your health. We’re going to help you help yourself by mandating that you use our pharmacy, or you can’t come back.“

Harold’s circumstances highlighted the difficulties that individual PLWH had in declining drug diversion when it is offered to them, but that health care settings and other systems can play a role in preventing diversion, ideally in a manner that is non-punitive and considers the systemic factors at play including poverty, as well as the role of substance use and mental health challenges.

### The COVID-19 pandemic exacerbated systemic impediments

The COVID-19 pandemic disrupted the health care system and lives of PLWH. We found themes regarding COVID-19 and its direct effects on mental health and substance use challenges, difficulties accessing therapy or substance use treatment during the early days of the pandemic, the pandemic’s contributions to social isolation, and finally the fact of living with HIV in the context of COVID-19 as a particular stressor. We found anxiety caused by the perceived increased vulnerability to COVID-19 by virtue of living with HIV was common. Some participants noted that their substance use increased during COVID, while for others, the fear of COVID reduced substance use patterns (“I’m good that it [COVID] happened. If [it] didn’t I still probably would be out there in the street ripping and running.”) Gerald, a 40-year-old Black, sexual minority, cisgender man who was diagnosed with HIV before age 18, went on to describe how social isolation combined with these anxieties to compound already existing depression:

I will not say that COVID did not have an impact on my emotions, but I was already having issues with my bipolar levels, depression issues. And I did find out that COVID did have an impact on me in some aspects. But COVID did make a lot of people isolated. And now you mention it, it did add to my depression. It did add to my sadness.

Restrictions on in-person activities and travel during the COVID-19 pandemic interfered with the ability to access needed substance use treatment. For example, in-person support groups were paused indefinitely, and some participants had to take on the management of substance use issues on their own. Many participants were in long-term recovery from substance use problems and described using substances periodically during COVID. Even when limited access to mental health services was possible, participants recalled being met with long wait times and numerous cancellations, especially for much needed in-person appointments. This was also the case with Samuel, a Black, sexual minority, cisgender man in his mid-30’s who was diagnosed with HIV when he was in his early 20’s:

I’ve been trying to see if I can go to a therapist, but these days they’re usually backed up and booked a month or two. […] And to schedule therapy it takes like three months. I mean that’s just to get the initial assessment…I want somebody in front of me, like somebody I can identify, and have some sense of accountability…in terms of you know, I can actually [see] the person I can go to. There’s something about having that visual person in front of you.

Accessing mental health care, substance use treatment, and health care was always a challenge for these participants located at the lowest socioeconomic strata, and COVID-19 made access even more difficult. Further, the pandemic aggravated some of the other factors that impeded HIV medication adherence prior to the pandemic; namely, social losses and social isolation, and also commonly led to increased substance use and mental health issues, particularly depression.

## Discussion

The public health system in the United States has set a goal to end the HIV epidemic by the year 2030 [40], but this aim will not be reached without eliminating racial/ethnic inequities in HIV care continuum engagement. Federal and local programs provide PLWH with HIV primary care, HIV medication, comprehensive care management, and supportive housing programs, with some variations in availability and access by geographical region [41–43], and many PLWH are eligible for federal income maintenance programs. Yet serious racial/ethnic inequalities in care continuum engagement and HIV health outcomes have persisted for decades, with African American/Black and Latino PLWH experiencing the lowest rates of engagement and the highest rates of morbidity and mortality compared to other racial/ethnic groups [1, 2]. This study took a qualitative approach to advance the literature on these inequities by uncovering and describing, from the perspectives of African American/Black and Latino PLWH with unsuppressed HIV viral load, the primary systemic factors that shape HIV management, how the factors operate, and how PLWH mitigate or manage them. Results included barriers well-documented in the literature such as poverty, along with new insights into how systemic barriers operate. Moreover, the study also uncovered and described factors less prominent in the literature, such as how the type and quality of housing placements can support or impede HIV management and on drug diversion. Participants were mainly long-term HIV survivors diagnosed with HIV 20 years prior, on average, and as such had extensive experiences managing HIV and taking HIV medication. They were enrolled in the project at a time when they were not taking HIV medication at all, or were taking it at levels insufficient to reach HIV viral suppression. In fact, those with unsuppressed HIV viral load are less likely to be involved in research compared to their peers who are consistently well-engaged along the HIV care continuum [44]. Thus, the qualitative approach was valuable in uncovering the perspectives of this under-studied subgroup of American/Black and Latino PLWH on a range of systemic barriers.

The study highlights the importance of viewing HIV management within the context of systemic factors, including chronic poverty, which shape participants’ options and opportunities for housing, meeting basic needs, social relationships, health care access, and physical and mental health. This is consistent with the growing consensus that the health of social groups is most strongly affected by systemic, rather than individual, factors [3]. One lens through which to understand the systemic barriers to the HIV care continuum identified in the present study is systemic racism. Similar to our definition of systemic and structural barriers to health presented above, systemic racism involves entire systems within society (legal, political, economic, health care, education, and criminal justice) [6]. Examples of systemic racism include impediments to home ownership, schools’ dependence on local property taxes, biased policing and sentencing of men and boys from minoritized backgrounds, environmental injustice, voter suppression policies, and residential segregation [6]. Structural racism is defined as the macro-level social forces, ideologies, institutions, and processes that interact with one another to generate and reinforce inequities among racial and ethnic groups [45]. Structural racism emphasizes how structures (i.e., laws, policies, institutional practices, and social norms) serve as the systems’ framework [6]. Systemic and structural racism are deeply embedded in laws, policies (both written and unwritten), and the deep-seated practices and beliefs that produce, tolerate, and propagate widespread unfair treatment and oppression of minoritized populations, with serious adverse health consequences [6]. Since systemic racism includes structural racism, we use the term systemic racism to refer to both concepts in this Sect. [6].

The larger literature highlights the role that systemic racism plays in HIV inequities. Bowleg and colleagues [46] recently called for ending systemic racism as an essential step to ending the HIV epidemic in the United States. They note recent studies demonstrate how inequalities entrenched in systemic racism such as incarceration, housing instability, police discrimination, neighborhood disadvantage, community violence, and poor access to social services, transportation, and childcare, serve as barriers to HIV prevention and treatment [46–48]. Many of these factors, which are systemically and culturally ingrained, affected the participants in the present study and had a real, tangible impact on their pathways for economic stability and health. Yet, national responses to the HIV epidemic inadequately address long standing socio-systemic issues. The national response to HIV such as the initiative to End the HIV Epidemic and the National HIV/AIDS Strategy have been criticized for failing to address systemic racism in efforts to end the HIV epidemic [46, 49]. Strategies that fail to recognize and address the role of systemic racism in the HIV epidemic risk reproducing patterns of inequalities that contribute to HIV disparities in prevention and treatment.

In the present study participants did not specifically point to systemic *racism* as a root cause of impediments to HIV management. Moreover, the constructs of race, racism, and discrimination were almost never mentioned in the interviews, even when participants were directly queried about them. We interpret these findings in part as indicating that systemic barriers are often “visible” to participants, as we found in the present study, but the connections between systemic barriers and systemic racism are not always apparent. Further, while it can be argued that systemic racism is pervasive among African American/Black and Latino PLWH, they may not commonly experience individual-level racism and discrimination in their everyday lives. In fact, past research found reports of racism and discrimination to be relatively low in this population [50]. It is also possible that participants in the present study experience individual-level racism and discrimination but declined to explore these factors with the researchers in this study. In a recent article, Adkins-Jackson and colleagues [51] provide guidance on new approaches to measure structural racism and recommend photovoice, a qualitative method for visually portraying experiences and sharing knowledge through photographs, along with life-course approaches [51].

We found participants experienced poverty as a fundamental cause of disengagement from the HIV care continuum, in part because it creates conditions that de-prioritize HIV and directly and indirectly interferes with HIV management. While the association between poverty and a lack of HIV viral suppression is well-documented in the literature [52–54], the present study provides PLWH’s perspectives on poverty’s adverse effects on their lives and how they attempt to mitigate it. Findings underscore and describe the complexity and difficulty of life in the lowest socioeconomic strata while managing HIV. For example, Maya’s lack of a working phone resulted in unfilled prescriptions for psychiatric medications which led to confusion and challenges accessing a food bank. HIV medications were not and could not be prioritized in this context. The multifaceted and siloed nature of social and health services impeded access to HIV care and reduced health and wellbeing. While HIV care services are generally centralized in a “medical home” model [55, 56], this population of African American/Black and Latino PLWH evidence need for a range of other resources and services such as for food, clothing, phone service, and mental health and substance use treatment services located outside HIV care settings. The present study highlighted the cascading nature of challenges rooted in poverty.

The COVID-19 pandemic complicated PLWH’s abilities to meet basic needs and access services, but these types of challenges were certainly present before the pandemic began. In past research we examined long-term HIV survivorship through the lens of symbolic violence, a type of nonphysical violence manifested in the power differential between social groups. We found that African American/Black and Latino PLWH are “ground down” over time by material, social, and emotional challenges and this leads to a sense of diminished self-worth and, at times, weakens the will to live, and also contributes to social isolation based in part on feeling devalued and dehumanized and the desire to avoid stigma [57]. Thus, systemic barriers affect PLWH directly, and their effects can be internalized over time, with deleterious effects.

Food insecurity is common among African American/Black and Latino PLWH [58, 59]. Financial benefit levels are not sufficient to prevent food insecurity in this population, and food insecurity is robustly related to unsuppressed HIV viral load [58]. Bowen and colleagues, for example, argue that systemic racism is a fundamental cause of food insecurity, because structural racism contributes to racial disparities in income and wealth, and because it is linked to food insecurity independent of poverty and socioeconomic status [60]. Further, racial discrimination is associated with food insecurity, along with living in states where stricter regulations and harsher punishments are tied to social assistance programs, including food assistance programs [60].

A recent scoping review found pharmacies in many geographical locations solicit the purchase of HIV medications, resulting in PLWH selling or diverting their medications [61]. Participants’ narratives underscore the tension between needing extra financial resources to survive but also wanting to take their HIV medication. Thus, participants commonly experienced themselves facing a set of mutually exclusive needs. Financial need combined with the opportunity to divert HIV medications are clearly major reasons why PLWH stop taking HIV medications in many cases.

Housing is critical to HIV management, and those without stable housing struggle to sustain HIV viral suppression, as has been well documented [62–64]. The present study yields insights into African American/Black and Latino PLWH’s experiences in different types of housing settings, and the effects of those settings on HIV management. Participants experience stable, independent housing as a necessary solid foundation from which to manage HIV and other aspects of their lives. Past studies have shown the benefits of congregate and supportive settings for PLWH on HIV adherence patterns [65, 66]. But in the present study, congregate and supportive settings were generally experienced as stressful, chaotic, lacking in support for personal autonomy, and denying dignity, since PLWH must follow strict rules and do not have private kitchens or bathrooms. Paradoxically, we found congregate settings can increase social isolation since interactions in these settings can be fraught or even largely transactional. Further, congregate settings can make it challenging for PLWH to reduce or eliminate hazardous alcohol and drug use, since substance use and the selling of drugs are common in these residences. On the other hand, congregate and supportive settings are certainly necessary and valuable for many PLWH [67, 68]. Taken together, these findings indicate the quality of housing is nearly as important as having housing itself [17], and that congregate and supportive housing is optimal for some, but not all PLWH [69, 70], underscoring the need for a flexible and individualized approach to housing placements, and more independent/private housing placements.

HIV care settings are critical to HIV management. We found that African American/Black and Latino PLWH appreciate their health care providers on the whole but that aspects of the settings and the care provided do not always sufficiently meet their needs. For example, it was common for PLWH to be thwarted by and frustrated with a health care system that they experienced as siloed and difficult to negotiate. Further, participants identified a range of characteristics of health care settings they did not find acceptable or helpful, including that PLWH are not always treated as experts on their own health, which we interpret as a lack of support for PLWH autonomy. Further, medical distrust was evident in the results, but results suggest that while providers may not have discouraged discussions of distrust, neither did they engage PLWH in discussions around distrust. But, distrust influences HIV medication patterns. Taken together we interpret these findings as indicating insufficient structural and cultural competency in settings. If settings or providers lack structural competency, they may fail to recognize ways in which PLWH’s reported symptoms, attitudes, or diseases may in fact be manifestations of systemic factors (e.g., related to health care delivery systems, housing policy, and federal entitlement benefit levels) that shape health and illness [27]. If they lack cultural competency, they may be insufficiently able to care for PLWH with diverse values, beliefs, and/or behaviors [28]. Systemic/structural and cultural factors were almost never explicitly addressed in health care and social service settings from participants’ perspectives, which may reduce satisfaction with care and inadvertently communicate to PLWH that poor engagement along the HIV care continuum is a problem caused by individual PLWH’s decisions and behavior.

Substance use is common among PLWH at non-hazardous and hazardous levels [71–73], and a factor that often complicated, and in some cases, impeded HIV care. We found HIV care providers are generally experienced as non-punitive and non-stigmatizing with respect to substance use. However, harm reduction, defined as a set of practical strategies and ideas aimed at reducing negative consequences associated with drug use, is lacking in HIV care settings from participants’ perspectives [74, 75]. Yet harm reduction is a critical aspect of substance use management for PLWH [76, 77]. Study findings suggest that acceptable and accessible support or treatment for substance use concerns were lacking, and PLWH were often located in residential settings that triggered use or were not effective at helping them optimally manage substance use. Participants commonly cycled through experiences of stress, loss, disruption, mental health symptoms, and substance use at hazardous levels, rooted in poverty.

Resilience is the capacity to withstand or to recover quickly from difficulties [78] and African American/Black and Latino PLWH clearly evidence resilience. Resilience is generally conceptualized as an individual or community-level attribute [79]. However, scholars have recently begun to question the construct of resilience in the context of systemic racism, arguing that how the term is applied can be biased, stigmatizing, and pathologizing [80]. The main argument is that racial/ethnic minority individuals and communities are expected to be resilient or thought to be in need of resilience support, but this framework does not consider larger socio-ecological structures and systemic racism that undermine individuals or communities [79, 80]. Although there may be an argument for the importance of resilience, ironically, the need for resilience tends to obscure the causal factors that deem it necessary in the first place [81].

### Limitations

The study has strengths such as methodological rigor, as well as limitations. First, it is focused on systemic barriers to engagement along the HIV care continuum and as such does not explore individual- and social-level factors in detail, but these types of factors, such as stigma, also serve as impediments to HIV management. Further, there are undoubtedly additional systemic factors that affect African American/Black and Latino PLWH that were not captured in this study. Another limitation is the possible influence of social desirability bias on findings. We sought to minimize social desirability bias during the interview process by asking general questions first and reminding participants they could and should feel free to decline to answer any question without penalty. The primary qualitative interviewers were not someone participants had previously met or worked with, as a further means of reducing social desirability bias. Although qualitative studies are not designed to yield generalizable results [82], the local context undoubtedly shaped findings. The study was carried out in New York City, located in the northeastern part of the United States. While federal programs provide resources to PLWH nationally, and local resources exist, overall, the northeast has more services for PLWH compared to other areas of the country [62, 83, 84]. Future studies can explore geographical differences in African American/Black and Latino PLWH’s perspectives on systemic barriers. The sample did not include monolingual Spanish-speaking participants, which limits the inferences we can make about the population of Latino PLWH as a whole. The sampling frame for the qualitative data; namely, purposive sampling for maximum variability, focused mainly on aspects of HIV (time since diagnosis, viral suppression), and we did not stratify by race/ethnicity or age. This limited our abilities to explore racial/ethnic differences or barriers among younger PLWH. As noted above, people at the lowest socioeconomic strata are over-represented in the population of PLWH. In the present study, all participant had indications of poverty but we do not have information on their socioeconomic trajectories (e.g., movement from higher to lower statuses or consistent low status). Future studies can examine such trajectories and their effects on HIV care continuum engagement. As we describe above, participants were influenced by multiple intersectional systems of oppression related to race/ethnicity, socioeconomic status, sex, sexual orientation, gender identity, and HIV status. The modest sample size did not allow us to examine results from an intersectional perspective, but future larger studies may permit this type of analysis. Further, we can draw on methods such as photovoice and life course perspectives, as described above, to explore systemic racism (as opposed to systemic barriers) with more precision [51]. Last, all participants in the present study received behavioral intervention components as part of the larger optimization trial, and their experiences with the trial are described elsewhere, but not in the present study [21].

**Fig. 1:**
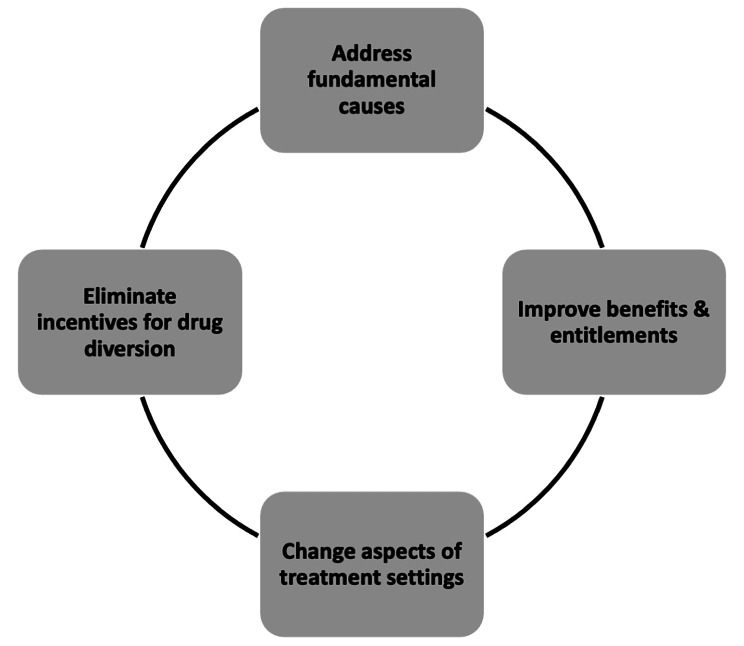
Schema of inter-related recommendations for policy, supports, and clinical practise based on the present study

### Implications of study fundings and recommendations

The present study yields implications for policy, research, and services, as summarized in Table 3; Fig. 1. Since poverty is a fundamental impediment to health and wellbeing among African American/Black and Latino PLWH, anti-poverty initiatives can be expanded and tailored to the needs of this population [85–87]. For example, Kalichman [88] argues that ending HIV hinges on reducing poverty. Social safety net programs for PLWH such as housing programs, income maintenance programs (“public assistance”), food security programs (e.g., food stamps), medical insurance, and drug assistance are critical and life-saving, but can be improved and expanded so they better meet the needs of PLWH [85]. For example, congregate settings are not optimal for all PLWH. Substance use is common among PLWH, but treatment programs are insufficient to meet need and may not be sufficiently culturally and structurally tailored to this population (e.g., autonomy supportive and harm reduction approaches may be lacking). Efforts to locate and shut down pharmacies engaging in drug diversion are needed, but particularly in conjunction with anti-poverty efforts for PLWH, since drug diversion provides vital financial resources to PLWH. As noted above, there is growing awareness that we cannot end the HIV epidemic in the United States without a meaningful and measurable commitment to addressing systemic racism as a core determinant of HIV [46]. Past research has found that African American/Black and Latino long-term HIV survivors have periods of sustained HIV viral suppression, but also commonly stop taking HIV medication in times of disruption, in part because chronic poverty does not allow for resources to buffer hardships [89]. It is vital to prevent disengagement from the HIV care continuum and locate and re-engage PLWH in times of disconnection.

**Table 3:** Inter-related recommendations for policy, supports, and clinical practice based on the present study

Address fundamental causes	
Poverty	Implement anti-poverty initiatives tailored to the needs of this population
Systemic racism	Address systemic racism in order to end the HIV epidemic
**Improve aspects of benefits & entitlements**	
Social safety net programs	Improve and expand programs for housing, income maintenance, and food security, as well as medical insurance and drug assistance programs in some geographical locations
Housing	The match between PLWH’s needs and the type and quality of housing matters. Place PLWH in private (non-congregate) settings as much as possible, as appropriate
	Increase income maintenance levels to support the stability of housing placements
**Change aspects of treatment settings**	
Structural/cultural competency	Improve structural and cultural competency in health care settings to enhance Black and Latino PLWH’s engagement in and satisfaction with care, trust in providers, self-disclosure, and health outcomes
Substance use	Provide autonomy-supportive, dignity-enhancing, and harm reduction approaches in HIV care settings to increase PLWH’s engagement, satisfaction, and treatment options
	Improve timely and easy access to substance use services, both in-person and virtual services
**Eliminate incentives to divert HIV medication**	
Drug diversion	Authorities can locate pharmacies engaging in illegal drug diversion and compel them to cease
	Increase PLWH’s income maintenance levels to reduce the need to divert HIV medications

## Conclusions

There is a saying that every system is perfectly designed to get the results it gets. Systemic factors drive serious and persistent racial/ethnic inequities in engagement along the HIV care continuum in the United States, despite substantial resources provided to treatment and programs for and research with PLWH. In the present study, African American/Black and Latino PLWH provided critical insights into these types of systemic barriers and how they operate. The larger literature argues systemic barriers in HIV must be considered in the context of systemic racism. Understanding and addressing the effects of systemic racism is necessary to dismantle its impact on health.

## Data Availability

Data are available upon reasonable request from the corresponding author.

## Abbreviations

HIV: Human immunodeficiency virus
PLWH: Persons living with HIV
